# Immunomodulation of NK, NKT and B/T cell subtypes in relapsed/refractory multiple myeloma patients treated with pomalidomide along with velcade and dexamethasone and its association with improved progression-free survival

**DOI:** 10.3389/fonc.2023.1271807

**Published:** 2023-12-04

**Authors:** Rao Prabhala, William E. Pierceall, Mehmet Samur, Lakshmi B. Potluri, Kevin Hong, Teresa Peluso, Srikanth Talluri, Angela Wang, Aishwarya Katiki, Sahan D. Vangala, Michael Buonopane, Vaishnavi Bade, Hannah Seah, Arthur Krogman, Sanika Derebail, Mariateresa Fulciniti, Suzan B. Lazo, Paul Richardson, Kenneth Anderson, Jill Corre, Herve Avet-Loiseau, Anjan Thakurta, Nikhil Munshi

**Affiliations:** ^1^ Department of Medical Oncology, Dana-Farber Cancer Institute, Boston, MA, United States; ^2^ VA Boston Healthcare System, Boston, MA, United States; ^3^ Harvard Medical School, Boston, MA, United States; ^4^ Bristol Myers Squibb, Summit, NJ, United States; ^5^ Department of Biostatistics, Harvard TH Chan School of Public Health, Boston, MA, United States; ^6^ Institut Universitaire du Cancer de Toulouse-Oncopole, Toulouse, France; ^7^ Oxford Centre for Translational Myeloma Research, Oxford, United Kingdom

**Keywords:** myeloma, pomalidomide, NK cells, B cells, and T cells

## Abstract

**Background:**

Multiple Myeloma (MM) patients exhibit dysregulated immune system, which is further weakened by chemotherapeutic agents. While cereblon-modulating agents, such as pomalidomide and lenalidomide, have been found to improve the immune profile, the efficacy of their impact in combination with other treatments is yet unknown.

**Methods:**

We conducted an immune-profiling of a longitudinal cohort of 366 peripheral blood samples from the CC4047-MM-007 (OPTIMISMM, NCT01734928) study. This study followed relapsed/refractory Multiple Myeloma (RRMM) patients who were treated with Velcade + dexamethasone (Vd), or Vd with pomalidomide (PVd). 366 blood samples from 186 patients were evaluated using multi-color flow cytometry at 3 timepoints: screening, day 8 of cycle 1, and cycle 3.

**Results:**

Among NK and NKT cell populations, adding pomalidomide showed no inhibition in the frequency of NK cells. When expression of double positivity for activation markers like, p46/NKG2D, on NK cells was higher than the median, PVd treated patients showed significantly better (p=0.05) progression-free survival (PFS) (additional 15 months) than patients with lower than the median expression of p46/NKG2D on NK cells. PVd treated patients who expressed CD158a/b below the median at cycle 1 demonstrated a significantly better PFS (more than 18months). Among B cell subtypes, PVd treatment significantly increased the abundance of B1b cells (p<0.05) and decreased Bregs (p<0.05) at day 8 of both cycle 1 and cycle 3 when compared to screening samples. Of all the B cell-markers evaluated among paired samples, a higher expression of MZB cells at day 8 of cycle 1 has resulted in enhanced PFS in PVd treated patients. Within T cells, pomalidomide treatment did not decrease the frequency of CD8 T cells when compared with screening samples. The higher the surface expression of OX-40 on CD8 T cells and the lower the expression of PD-1 and CD25 on CD4 T cells by PVd treatment resulted in improved PFS.

**Conclusion:**

The prognostic significance for the number of immune markers is only seen in the PVd arm and none of these immune markers exhibit prognostic values in the Vd arm. This study demonstrates the importance of the immunomodulatory effects and the therapeutic benefit of adding pomalidomide to Vd treatment.

## Introduction

MM patients generally display worsening PFS upon successive relapse (1, 2), potentially due to the loss of immunocompetency, especially considering that fact that MM cells are responsible for a dysregulated immune system (3). Plasmacytoid DCs (pDCs) and Th17 cells are more abundant in MM patients and support MM cell growth (4–6). In addition, invariant NK cells (7) are dysfunctional in myeloma, and MM patients have a higher frequency of myeloid-derived suppressor cells (MDSC) (8) and an elevated ratio of B2 cells to B1 cells. Overall, patients tend to have weakened immune systems, as evidenced by very low levels of anti-hepatitis antibodies following vaccination. This may explain why, when myeloma patients are vaccinated with HLA-A2-restricted multi-peptides (XBP-1, CD138, and CS-1), the generated CTL-memory cells are unable to produce appreciable clinical responses against tumor cells (9). In addition, despite the rapid clinical response MM patients have in response to BCMA-CAR-T cell therapy, many patients still relapse (10), perhaps due to deficits in the immune compartment.

Immunomodulatory agents (IMiDs) and proteasome inhibitors in combination with corticosteroids are standard-of-the-care in newly diagnosed multiple myeloma (NDMM) and RRMM patients. However, many of these agents dampen immune responses. Dexamethasone is a steroid that reduces inflammation, whereas velcade is an inhibitor of the ubiquitin-proteasome proteolytic pathway and thus can affect the turnover of lymphocyte receptors, ligands, cytokines, and chemokines. Though velcade’s activity is a mixture of immunosuppressive and immunostimulatory effects (11), combining it with dexamethasone results in generally immunosuppressive changes. Despite this, Vd is an effective treatment option. The effectiveness of Vd treatment is further increased when combined with pomalidomide, PVd, pushing PFS from 7.1 months in Vd treatment to 11.2 months by PVd treatment in RRMM patients (12). As such, pomalidomide-based triplet regimens are a safe and effective standard of care in RRMM patients.

It has been postulated that the clinical benefit of PVd is derived from enhanced immunocompetency (13–15). Pomalidomide targets Cereblon, which is part of the CRL4 E3 ubiquitin ligase, leading to the degradation of Ikaros and Aiolos, which are T cell repressors (16–19). However, due to the complex effects of each drug, it is impossible to say for certain, the exact changes that occur in the immune compartment by these drugs without clear understanding. The results from this study would be critical to not only confirming pomalidomide’s immunostimulatory effects but also establishing how the changes are happening in the immune compartment following therapy, which could be informative for improving immunotherapy strategies.

Here, we characterized the immune changes in RRMM treated with PVd vs Vd in the OPTIMISMM trial to understand the immunomodulatory effects of pomalidomide. We assessed peripheral blood samples taken at three time points from each treatment with multi-color flow cytometry to investigate immune subpopulations and the expression of their surface markers. This is the first time such a large (N=366 from 186 RRMM patients) sample set has been used for the evaluation of immune biomarkers in a clinical setting. When comparing the two treatment arms, PVd restored some immune cell subpopulations that counterbalanced the immunosuppression observed in Vd-treated patients. These results are especially notable as the immune enhancements driven by PVd were observed in patients relapsing or refractory to a previous immunomodulatory agent treatment (lenalidomide). These results establish the immunostimulatory mechanisms of pomalidomide when combined with dexamethasone and velcade, likely contributing to its clinical response benefits in RRMM patients.

## Materials and methods

### Study design and patient cohort

**Figure 1:**
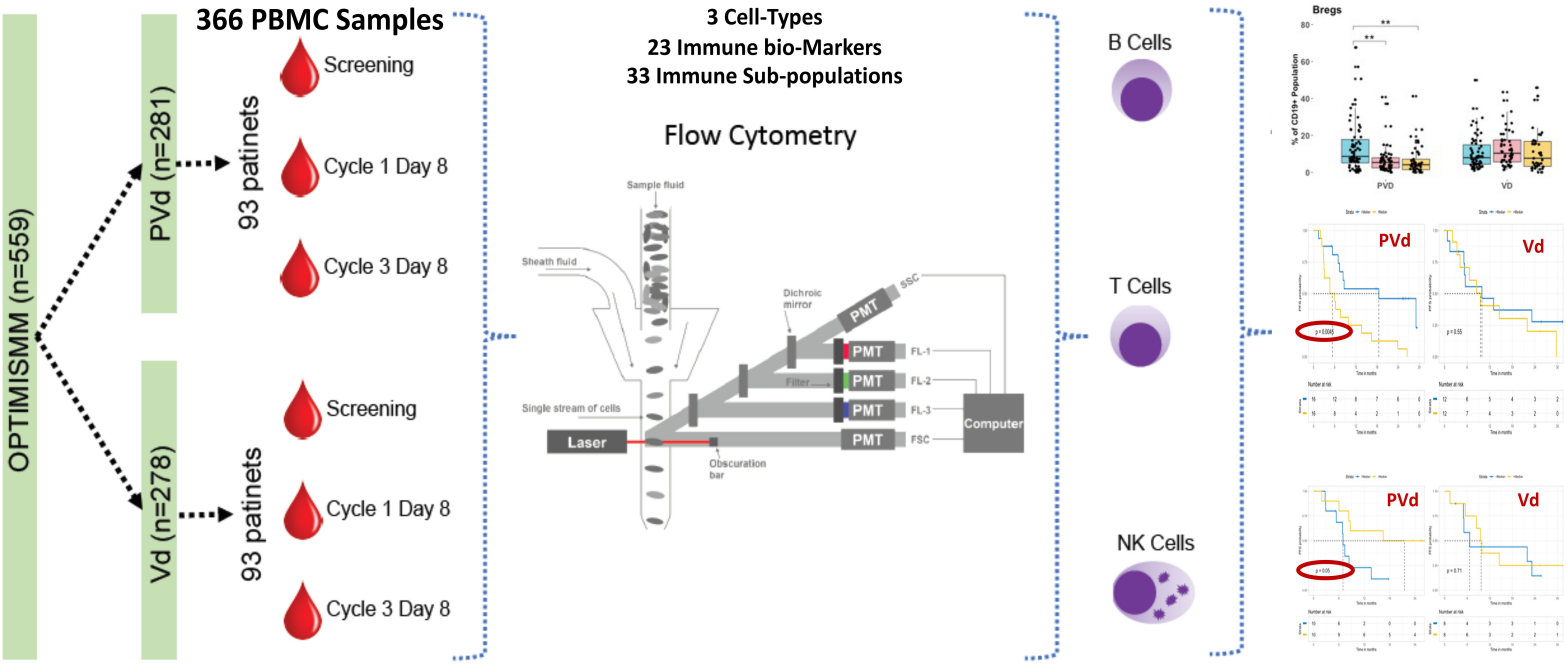
Current investigation schema and workflow. Peripheral blood samples (n=366) were collected from the OPTIMISMM trial from screening and on treatment cycle 1 day 8 and cycle 3 day 8. Of 559 patients treated with PVd or with Vd, immune profiling was done using 186 patients. There were no significant differences between clinical and immune profiling patients in terms of demographics or clinical parameters. Flow cytometry employed 3 panels comprising 23 unique markers to capture 33 immune populations for deep characterization of immunophenotyping subsets.

Three hundred and sixty-six (366) peripheral blood samples were collected at screening, cycle 1 day 8 and cycle 3 day 8 from one hundred and eighty-six (186) patients enrolled in study CC4047-MM-007 (OPTIMISMM), a phase 3 open-label randomized (PVd versus Vd treatments) clinical trial (ClinicalTrials.gov NCT01734928) (12) and were analyzed by multi-color flow cytometry. Again, this is the first time such a large sample set has been analyzed for MM patients. Patients have balanced clinical/pathological characteristics between two treatment arms (N=93 for PVd, N=93 for Vd) as shown in **Figure 1**; **Tables 1**–**4** for the explanation of study workflow and patient demographics, respectively. This study was approved by the institutional review board or ethics committees. All patients provided written informed consent. The study was executed in accordance with the principles of the Declaration of Helsinki. The investigators designed the study in conjunction with the sponsor, Bristol Myers Squibb.

**Table 1:** Comparison of demographics of the patients used in both clinical trial and immune study.

	PVd		Vd	
	Trial(n=281)	Immune Study(n=95)	Trial(n=278)	Immune Study(n=95)
**Female**	126(44.8%)	45(47.4%)	131(47.1%)	43(47.2%)
**Male**	155(55.2%)	50(52.6%)	147(52.9%)	48(52.8%)
**Age**	67[29-87]	68[38-87]	68[27-89]	69[39-89]

**Table 2:** Comparison of high risk and translocations of patients used in both clinical trial and immune studies.

	PVd						Vd					
	Trial(n=281)			Immune Study(n=95)			Trial(n=278)			Immune Study(n=95)		
	Yes	Evaluation	%	Yes	Evaluation	%	Yes	Evaluation	%	Yes	Evaluation	%
**High Risk**	61	198	30.81%	16	67	23.88%	49	181	27.07%	12	52	23.08%
**del17p**	28	190	14.74%	10	64	15.63%	21	172	12.21%	4	48	8.33%
**t(4;14)**	37	254	14.57%	7	88	7.95%	29	228	12.72%	8	73	10.96%
**t(14;16)**	4	224	1.79%	2	75	2.67%	7	199	3.52%	1	63	1.59%
**del13q**	66	169	39.05%	21	59	35.59%	48	158	30.38%	17	44	38.64%
**del1p**	21	201	10.45%	8	69	11.59%	13	181	7.18%	4	50	8.00%
**gain1p**	86	201	42.79%	30	69	43.48%	73	181	40.33%	23	50	46.00%

**Table 3:** Comparison of prior-treatments of the patients between clinical trial and immune study.

	PVd						Vd					
	Trial(n=281)			Immune Study(n=95)			Trial(n=278)			Immune Study(n=95)		
	Yes	Evaluation	%	Yes	Evaluation	%	Yes	Evaluation	%	Yes	Evaluation	%
**Lenalidomide**	281	281	100.00%	95	95	100.00%	278	278	100.00%	91	91	100.00%
**Valcade**	201	281	71.53%	69	95	72.63%	203	278	73.02%	66	91	72.53%
Refractory*	200	281	71.17%	71	95	74.74%	191	278	68.71%	63	91	69.23%

*to lenalidomide.

**Table 4:** Comparison of clinical characteristics of the patients between clinical trial and immune study.

	PVd						Vd					
	Trial(n=281)			Immune Study(n=95)			Trial(n=278)			Immune Study(n=95)		
	Yes	Evaluation	%	Yes	Evaluation	%	Yes	Evaluation	%	Yes	Evaluation	%
**Transplant**	161	281	57.30Z%	54	95	56.84%	163	278	58.63%	53	91	58.24%
**ISS I at entry**	149	281	53.02%	44	95	46.32%	138	278	49.64%	49	91	53.85%
**ISS II at entry**	85	281	30.25%	34	95	35.79%	90	278	32.37%	27	91	29.67%
**ISS III at entry**	47	281	16.73%	17	95	17.89%	50	278	17.99%	15	91	16.48%
**IgG**	193	278	69.42%	64	95	67.37%	185	269	68.77%	56	82	68.29%
**IgA**	58	278	20.86%	20	95	21.05%	56	269	20.82%	26	82	31.71%
**IgM**	2	278	0.72%	0	95	0.00%	0	269	0.00%	0	82	0.00%
**Light chain Kappa**	12	281	4.27%	5	95	5.26%	16	278	5.76%	5	91	5.49%
**Light chain Lambda**	13	281	4.63%	5	95	5.26%	10	278	3.60%	2	91	2.20%
**Median PFS**	11.2 Months			11.2 Months			7.1 Months			6.3 Months		

### Treatment

Patients received PVd or Vd in 21-day cycles until disease progression or unacceptable toxicity. Velcade was administered intravenously (IV) or subcutaneously (SC) at 1–3 mg/m (2) twice weekly for the first two weeks of cycles 1–8, then once per week for the first 2 weeks of the 21-day cycle thereafter. Dexamethasone, at a dose of 20 mg/day (10 mg/day for patients >75 years of age), was given twice orally, first, on the same day as velcade and again on the following day. Dose interruptions and reductions were permitted throughout the study. In patients eligible to receive pomalidomide, 4 mg/day was administered daily orally, from days 1 to 14 of each 21-day cycle.

### Flow cytometry

Peripheral blood mononuclear cells (PBMCs) from blood samples collected in heparin tubes were isolated by Ficoll-Paque Plus (GE Healthcare) density centrifugation as described^,6^ and million cells were used to stain with fluorochrome conjugated monoclonal antibodies according to manufacturing company recommendations (Biolegend – 5ul per million cells in 100 ul volume of stain buffer) using 100 ul of brilliant stain buffer (BD Biosciences) for 20 minutes at room temperature. Following incubation with antibodies, cells were washed with PBS, then fixed with 0.5ml fixation buffer (BD Biosciences). Samples were analyzed using BD LSR Fortessa. Initial gating included removal of debris (FSC-A^low^ events) and gating on singlet FSC and SSC populations, followed by lymphocytes gating (adjusted to include T cells, B cells, NK cells). The antibodies used in this study were purchased from Biolegend and the information on these antibodies, including conjugated fluorochromes, catalogue numbers, clone numbers, lasers and filters is provided in **Table 5**.

**Table 5:** List immune biomarkers assessed by multi-color flow cytometry.

		Biolegend			
Cells/Surface Markers	Conjugation Dyes	Cat#	Clones	Laser(nm)	Filter
NK Cells					
**CD56 (NCAM)**	BV-605	318334	HCD56	Violet (405)	610/20
**NKG2D-CD314**	APC-Cyanine 7	320824	1D11	Red (640)	780/60
**p46-CD335**	AF-700	331932	Clone 9E2	Red (640)	710/50
**CD159a-NKG2A**	FITC	375128	S19004C	Blue (488)	530/30
**p75-CD328-SIGLEC7**	APC	339206	6-434	Red (640)	660/20
**CD158a-KIR2DL1**	BV-711	583183	HP3E4	Violet (405)	710/20
**CD158b-KIR2DL2**	PE-Cyanine 7	312610	DX27	Yellow-green (561)	780/60
**CD3**	BV-421	317344	OKT3	Violet (405)	450/50
T cells					
**CD3**	BV-605	317322	OKT3	Violet (405)	610/20
**CD4**	PE-Cyanine 7	317414	OKT4	Yellow-green (561)	780/60
**CD8**	APC-Cyanine 7	344714	SK1	Red (640)	780/60
**CD45RA**	FITC	304106	HI100	Blue (488)	530/30
**CD197-CCR7**	BV-510	353232	G043H7	Violet (405)	525/50
**CD134-OX-40**	APC	350008	Ber-ACT35	Red (640)	660/20
**CD25-IL-2R**	PE	385608	S20019C	Yellow-green (561)	582/15
**CD279-PD-1**	BV-421	367422	NAT105	Violet (405)	450/50
B cells					
**CD23**	FITC	561146	M-L233	Blue (488)	530/30
**CD19**	BV605	302244	HIB19	Violet (405)	610/20
**CD27**	PE-Cyanine 7	302838	O323	Yellow-green (561)	780/60
**GM-CSF**	BV421	562930	BVD2-21C11	Violet (405)	450/50
**CD5**	PE	300608	UCHT2	Yellow-green (561)	582/15
**CD43**	APC	343206	CD43-10G7	Red (640)	660/20
**CD185 (CXCR5)**	BV510	563105	RF8B2	Violet (405)	525/50

### Statistical methods

Statistical analyses were performed using the R programming language. The significance between groups like screening vs cycle 1 or screening vs cycle 3, was obtained by adjusted p-values from Student “t” test analysis using the R programing language. The number of stars indicates the level of p-value significance (*p<0.05; **p<0.01; ***p<0.001; and ****p<0.0001) between groups including, screening vs cycle 1; screening vs cycle 3 and cycle 1 vs cycle 3 for each marker in each treatment arm. The Wilcoxon rank-sum test was used to compare two independent groups at three timepoints for each marker and a Kruskal–Wallis test was used to compare timepoints for each treatment arm. False discovery rates (FDR) were then calculated as q-values from raw p-values to adjust for multiple comparisons in our biomarker analysis. All graphical representations were generated using the ggplot2 and ggpubr libraries in R. The Cox proportional hazard model was used for univariate and multivariate survival analysis. For each immune marker, patients were dichotomized by median expression among all patients. The log rank test was used to assess the significance of pharmacodynamic changes between timepoints unless indicated otherwise. The change from screening for each pharmacodynamic parameter was calculated as the difference between the post-treatments (day 8 of cycle 1 and 3) and pre-treatment (screening) data.

## Results

Three hundred and sixty-six (366) peripheral blood samples were collected at screening, cycle 1 day 8 and cycle 3 day 8 from one hundred and eighty-six (186) patients (**Figure 1**) enrolled in CC4047-MM-007 (OPTIMISMM) study, a phase 3 open-label randomized (PVd versus Vd treatments) clinical trial (ClinicalTrials.gov NCT01734928) (12). Patients showed balanced clinical/pathological characteristics between two treatment arms for (N=93 for PVd, N=93 for Vd, **Tables 1** **–** **4**). Patient characteristics were compared between clinical and immune studies as shown in **Tables 1** **–** **4**. The patient demographics (sex and age) in percentages are presented in **Table 1**. There were no significant differences in percentage of females and males present in the study; or the age of patients used in both clinical and immune studies. In regards to high-risk factors and chromosomal translocations, including del17p, t (4;14), t(14;16), del13q, del1p and gain1p, there were no differences seen between clinical and immune studies (**Table 2**). In terms of prior lines of treatments, there were no significant differences in the percentage of patients who were treated or refractory to lenalidomide and/or velcade prior to this study (**Table 3**). There were no deviations observed from clinical characteristics of patients, including ISS staging (I, II and III), prior transplantation, type of myeloma (IgG, IgA, and IgM in addition to light chains) and the length of PFS in both studies (**Table 4**). As there were no significant differences between patients used in the clinical trial and in this immune study, as indicated in **Tables 1** **–** **4**; these results indicate that patient’s PFS and the changes seen in the frequency of immune cells and the expression of their surface cell-markers, appeared to be due to the influence of their respective treatment arm, PVd or Vd.

This immune study evaluated the expression of surface markers on three different cell-types, including NK/NKT, B, and T cells using multicolor flow cytometry. This is the first immune study which utilized such a large sample-set (366 samples from 186 patients) to evaluate the frequency of immune markers and their association with PFS following the OPTIMISMM clinical response trial (N=559). The primary goal of the study was to evaluate immune marker changes following PVd or Vd treatments. Often the relative expression of a given surface marker on a given cell-type may not be significantly different. However, where the expression falls higher or lower than the median level for a given surface maker on given cell-type may have impacted the clinical response and, ultimately, a patient’s PFS following PVd treatment. This impact on survival was likely due to the functional abilities of that immune cell, their interactions with other immune cell-types, and finally, their interactions with MM tumor cells.

### Pomalidomide effect on NK cells and their association with clinical outcome benefit

**Figure 2:**
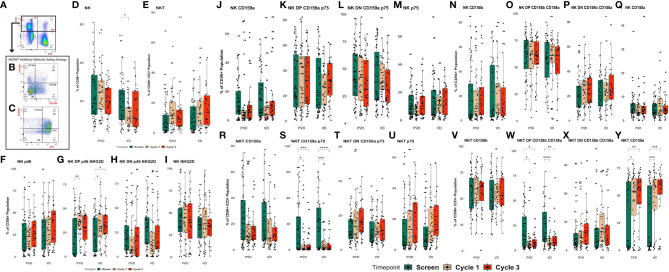
Pharmacodynamic changes in patients’ peripheral NK and NKT cell subpopulations following treatment. Peripheral blood specimens taken from patient’s pre-treatment (screening) or following administration of PVd or Vd were assessed for NK and NKT cell subpopulations. Representative gating strategy for NK and NKT cells **(A–C)**, the expression of CD56+ NK cells and CD56+CD3+ NKT cells **(D–E)**, the expression of NKG2D and p46 on NK cells **(F–I)**, the expression of suppressive markers on NK cells, including CD159a and p75 **(J–M)** and CD158a and b **(N–Q)**, and the expression of suppressive markers on NKT cells, including CD159a and p75 **(R–U)**, CD158a and b **(V–Y)**. The number of stars indicates the level of p-value significance (*p<0.05; **p<0.01; ***p<0.001; and ****p<0.0001) between groups including, screening vs cycle 1; screening vs cycle 3 and cycle 1 vs cycle 3 for each marker.

To evaluate the impact of PVd or Vd regimens on the pharmacodynamics of NK and NKT cells, peripheral blood specimens from patients, taken pre-treatment (screening) or following administration of treatment, at day 8 of cycles 1 and 3, were assessed by flow cytometry for NK and NKT cell subpopulations, their activation, and expression of suppression markers. Specifically, activation markers p46/CD335 and NKG2D/CD314, and suppressive markers (KIRs including CD159a/NKG2A, p75/CD328, CD158a/KIR2Dl1 and CD158b/KIR2DL2/3) were assessed. A representative gating strategy for NK cells, CD56+ NK cells and CD56+CD3+ NKT cells is shown in **Figures 2A–C**. In general, the gated lymphocyte population was sub-gated initially on expression of CD56 and CD3 to identify NK cells (CD56+) and NKT cells (CD56+CD3+) as shown in **Figure 2A**. Both cell-types were further sub-gated using CD158a and CD158b monoclonal antibodies to obtain CD158a+, CD158a+/b+, CD158b+, and CD158a-/b-; as shown in **Figure 2B**. Similarly, NK and NKT cells, were sub-gated by CD159 and p75 monoclonal antibodies to obtain single-positive-CD159a+, double-positive-CD159a+p75+, double-negative-CD159a-p75-, and single-positive-p75+ populations as shown in **Figure 2C**. Additionally, the expression if activation markers, like p46 and NKG2D on NK cells were obtained from sub-gated NK cells. Significances were shown as adjusted p-values between the two groups (PVd vs Vd) and were calculated using Student’s “t” test via the R programing language. Overall, the pomalidomide containing regimen (PVd) had the most significant positive effects on NK and NKT cells compartments. In the Vd treatment arm, the percentage of CD56+ NK cells were significantly lower (p<0.05) at cycle 1 day 8 compared with screening; however, no significant changes were seen in PVd treatment arm (**Figure 2D**). NKT cells did not show a significant change in either PVd-treated or Vd-treated patients (**Figure 2E**).

The number of double positive for NKG2D/p46, or activated NK cells, were significantly increased from screening to cycle 1 (p<0.01) for PVd treated patients, and between screening to cycle 1 and cycle 3 (p<0.05) for Vd treated patients (**Figure 2G**). However, the number of p46+ NK cells (**Figure 2F**), double negative NKG2D/p46 NK cells (**Figure 2H**), and NKG2D+ NK cells (**Figure 2I**) were not significantly changed between timepoints in either treatment arm. The percentage of CD56+ NK cells expressing different KIR molecules (CD159a+, CD159a+/p75+, CD158a-/p75- and p75+) (**Figures 2J–M** respectively) (CD158b+, CD158a+/b+, CD158a-/b- and CD158a+) (**Figures 2N–Q** respectively) also did not show a significant change between timepoints for both treatments of PVd-treated or Vd-treated patients. More importantly, NKT cells expressing double positivity of suppressive CD159a and p75 KIR molecules were significantly decreased between screening and day 8 of cycle 1 (p<0.05), and cycle 3 (p<0.001) following PVd treatment and between screen and day 8 of cycle 1 (p<0.001), and cycle 3d (p<0.0001) following Vd treatment (**Figures 2S**). However, similar decreases were not seen in NKT expressing single positive CD159a (**Figure 2R**), double negative CD159a/p75 (**Figure 2T**), and single positive p75 (**Figure 2U**) in either arm. The percentage of NKT cells expressing double positive KIR molecules CD158a and CD158b (**Figure 2W**) were significantly reduced, while expression of single positive CD158a NKT cells (**Figure 2Y**) were significantly increased in both treatment arms. The expression of CD158b+ (**Figure 2V**), and double negative CD158a-/b- (**Figure 2X**) on NKT cells was not altered in either treatment arm. This could have a positive impact, as myeloma cells are sensitive to lysis by NKT cells (20).

**Figure 3:**
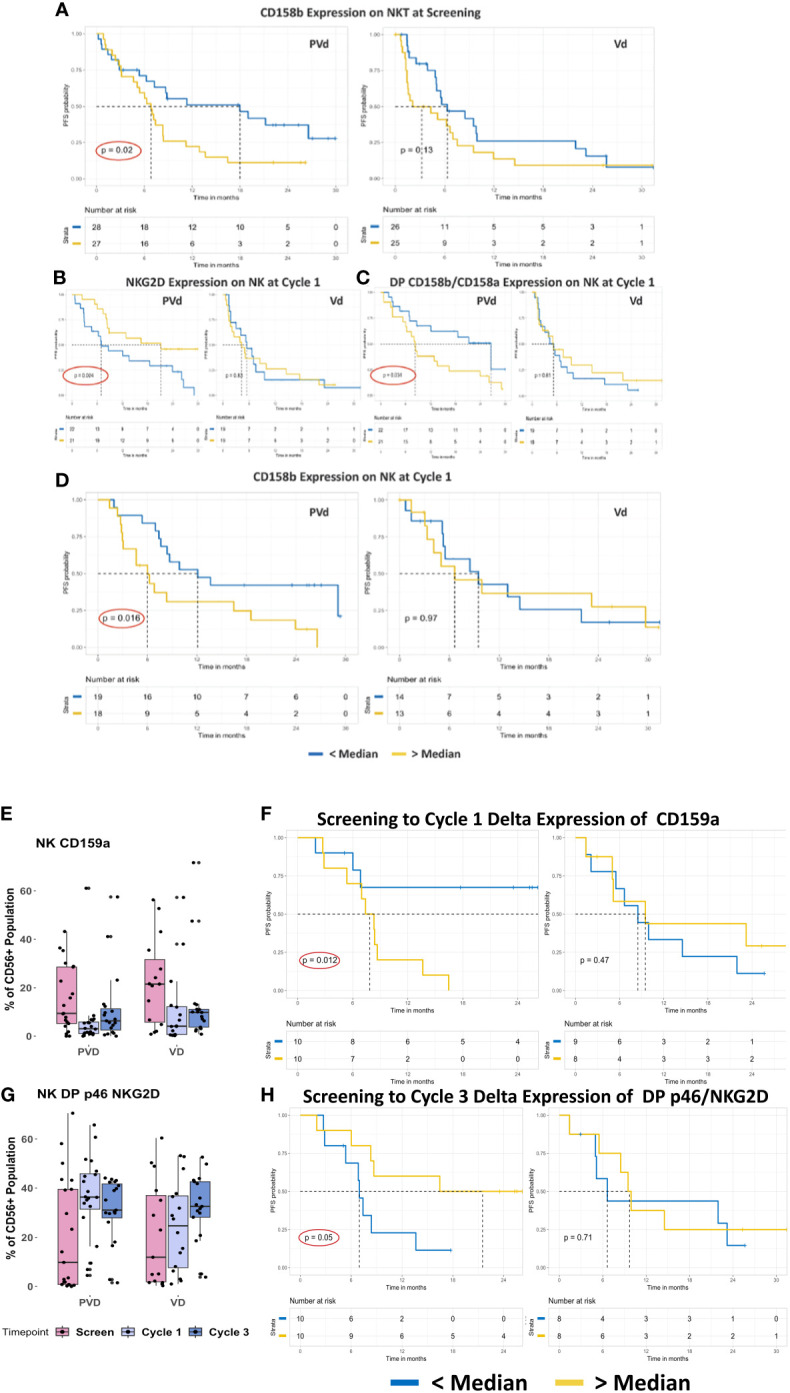
Representative baseline and post-treatment NK/NKT cells associated with favorable PFS in PVd–treated patients. PVd-treated patients showed association of immune cell subpopulation levels at pre-treatment and post-treatment at cycle 1 and 3 day 8 **(A–H)**. The expression of CD158b on NKT cells, a suppressive KIR molecule **(A)**, the expression of NKG2D, an activation marker, on NK cells **(B)**, the frequency of NK cells expressing CD158a+/b+ **(C)**, and expression of CD158b on NK cells **(D)**, is associated with significantly improved PFS by PVd. The expression of CD159a on NK cells from samples paired between screening and cycle 1/3 **(E)** and their effect in terms of PFS **(F)** is shown. The number of NK cells that are double-positive for p46 and NKG2D **(G)**, and the PFS association of the frequency of NK cells with their expression at cycle 3 relative to screening in paired samples are shown **(H)**. The Kaplan-Meier survival plots are drawn for each arm separately for the expression of a given immune marker (CD158b-NKT, NKG2D, CD158a/b, CD158b, CD159a and p46/NKG2D on NK) expression below median (blue dark line) and above median (yellow light line).

It is not easy to identify significant differences in the frequency of NK/NKT cells, even with a large sample set, as treatments may not affect the expression of a given surface marker on NK or NKT cells. However, their functional capacity and their interactions with other immune cells in the bone-marrow (BM) microenvironment and with myeloma cells may influence the survival of patients. As such, we investigated the association of expression differences of immune markers to patient survival, particularly PFS, with each treatment arms between three timepoints - screening, and post-treatment at day 8 of cycle 1 and cycle 3 (**Figures 3A–H**). For each immune marker among the three studied cell-types (NK/NKT, B, and T cells), patients were ranked by range of expression, then dichotomized into greater-than and lower-than median-expression cohorts. The log rank test was used to assess the significance of pharmacodynamic changes between timepoints within each arm.

Patients who expressed CD158b on NKT cells below the median level at screening demonstrated an improving tendency in PFS in both arms. However, only PVd patients who expressed CD158b below the median level on NKT cells at screening showed a statistically significant improved PFS (p=0.02) compared to patients who expressed CD158b above the median level on NKT cells (**Figure 3A**). Though a lower expression of CD158b following Vd is not statistically significant, PFS was significantly improved by the addition of pomalidomide. PVd treated patients achieved over 12 months of additional PFS, suggesting that CD158b can be used as a predictive immune marker at screening.

NKG2D is an activation marker expressed on NK cells and its expression indicates the activation status of NK cells. In PVd patients, when NKG2D was found to be expressed higher than the median compared with patients who expressed NKG2D lower than the median in cycle 1, an improved PFS of an additional 14 months was observed (p=0.024) (**Figure 3B**). If NK cells at cycle 1 exhibited lower than the median expression of CD158a+b+, a favorable PFS was observed (p=0.034) in PVd patients (additional 18 months) compared to patients who expressed higher than median levels of CD158a/b (**Figure 3C**). As such, NKG2D and double-positive CD158a/b on NK cells may also serve as early prognostic markers in PVd treated patients. When CD158b expression on NK cells is lower than the median at cycle 3, PVd treated patients showed significantly (p=0.016) better PFS (additional 6 months) compared with patients with higher than the median expression of CD158b on NK cells (**Figure 3D**). This immune marker, CD158b, on NK cells may aid as a late prognostic indicator for PVd treatment. This type of survival association for the above mentioned immune markers on NK cells was not seen in patients on the Vd treatment arm.

Expression of CD159a, a KIR suppressive marker on NK cells, was observed to have a declining trend from screen to Cycle 1 and 3 in both arms (**Figure 3E**) among paired samples, though it was not statistically significant. To understand if changes in NK/NKT cell marker expression following treatment affects PFS, survival analysis was conducted using delta values which referred to the difference in KIR expression between pre-treatment (screening) and post treatment (cycle 1 and cycle 3). PVd patients with lower than median delta expression of CD159a (blue darker line in **Figure 3F**) had significantly (p=0.012) better PFS (additional 24 months) when compared with a higher than median expression of CD159a (yellow lighter line in **Figure 3F**). Higher frequency of double positive p46/NKG2D-expressing NK cells than the median (yellow lighter line in **Figure 3H**) showed significantly better (p=0.05) PFS (additional 15 months) for PVd patients when compared to a lower than the median frequency (blue darker line in **Figure 3H**). These type of survival associations for above mentioned immune markers was not seen in Vd treated patients.

These results suggested that the addition of pomalidomide to Vd treatment exhibited therapeutic benefit on survival and clinical response by increasing activation markers, like NKG2d alone or in combination with p46; and by reducing immune suppression due to expression of KIR molecules, like CD158b alone, CD158a/b and CD159a, on NK cells in patients. These results on NK cells indicate that PVd treatment had a positive influence on NK cells to provide an improved anti-myeloma response.

### Pomalidomide has varying effects across B cell subtypes and their association with clinical outcome benefit

**Figure 4:**
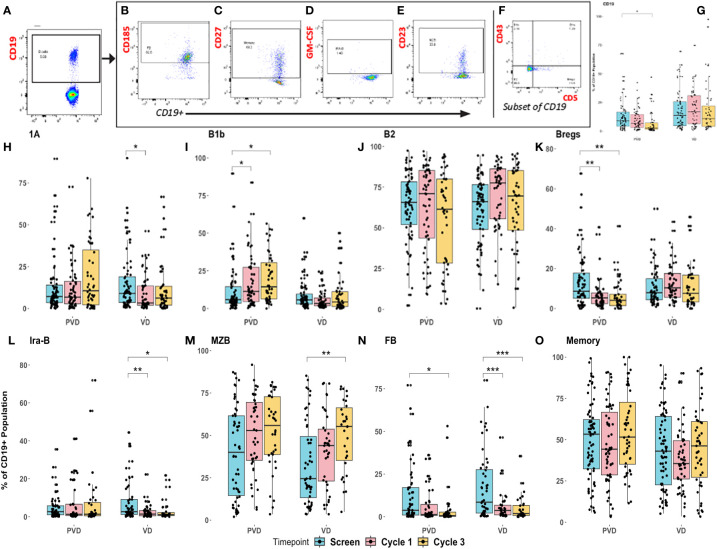
Pharmacodynamic changes in patients’ peripheral immune B–cell subpopulations following treatment with PVd versus Vd. Peripheral blood specimens from patients were assessed for pharmacodynamic changes in B-cell immune subpopulations by multi-color flow cytometry. Representative strategy was shown for gating of 8 different sub-populations of CD19+ B cells **(A)**, follicular B cells-FB, measured by CD185 **(B)**, memory B cells measured by CD27 **(C)**, Ira-B cells measured by GM-CSF **(D)**, MZB measured by CD23 **(E)**, and 4 sub-sets of B cells (B1b, B1a, B2 and Bregs) **(F)**. Changes depicted here include Pomalidomide with Vd-regimen representative specific enhancements panel **(G)** B cells, panel **(H)** B1a B cells, panel **(I)** B1b B cells, panel **(J)** B2 B cells, panel **(K)** Bregs, panel **(L)** Ira-B cells, panel **(M)** MZB cells, panel **(N)** FB cells, and panel **(O)** memory cells. Pharmacodynamic trends were noted in cycle 1 day 8 and cycle 3 day 8 specimens relative to screening. MZB, Marginal zone B cells; FB, Follicular B cells that are matured in germinal centers in the spleen and lymph-nodes. The number of stars indicates the level of p-value significance (*p<0.05; **p<0.01; ***p<0.001) between groups including, screening vs cycle 1; screening vs cycle 3 and cycle 1 vs cycle 3 for each marker.

Peripheral blood specimens from screening or post-treatment (cycle 1 day 8 and cycle 3 day 8) following administration of PVd or Vd were assessed for pharmacodynamic changes in 8 B-cell immune subpopulations by multi-color flow cytometry. The representative subpopulation gating strategy for the 8 different sub-populations of CD19+ B cells are shown in **Figures 4A–F**. Initially, CD19+ B cells are gated from the lymphocyte gate, as shown in **Figure 4A**. From this CD19+ B cell gate, via measurement of the expression of CD185, we identified follicular B cells (FB), which produced immunoglobulins and B cell-receptor diversity (**Figure 4B**); via expression of CD27, we identified memory B cells (**Figure 4C**); via expression of intra-cellular GM-CSF, we identified innate response activator B cells (IRA-B), derived from B1a population that are important in bacterial clearance (**Figure 4D**); and, finally, via expression of CD23, we identified the marginal zone B cells (MZB), which are important provide immunity against blood-borne pathogens (**Figure 4E**). Additionally, CD19+ B cells were further categorized into four important main subsets by the expressions of CD43 and CD5. The four different B cell sub-sets (**Figure 4F**) included B1b (CD19+CD43+CD5-), which provide protection against acute bacterial infections and are integral to vaccine responses; B1a (CD19+CD43+CD5+), which is considered to be natural antibody-producing cells present from birth; B2 (CD19+CD43-CD5-), which produce all types of immunoglobulins and complete B cell-receptor diversity; and, lastly, Bregs (regulatory B cells) (CD19+CD43-CD5+), which are much more potent in the regulation of both T and B cell-responses, producing cytokines such as IL-10 which upregulate T regulatory cells (Tregs).

PVd treatment was associated with a significant decrease in the overall number of B cells (CD3-CD19+) (**Figure 4G**). B1a cells are considered innate, long-lived, and self-renewing natural antibody-producing (IgM) B cells with a limited repertoire and without memory. B1a cells (CD19^+^CD43^+^CD5^+^) were significantly reduced in Vd treated patients but not in PVd treated patients (**Figure 4H**). B1b cells (CD19^+^CD43^+^CD5^-^) are important in providing protection against acute bacterial infections and producing vaccine responses (21, 22). In this study, we observed that B1b cells were progressively increased at cycle 1 day 8 and cycle 3-day 8 following PVd treatment, while Vd B1b cell-populations remained unaltered (**Figure 4I**).

B2 cells are important for producing the most of the body immunoglobulins and we did not observe any changes in their frequency following either treatment (**Figure 4J**). Bregs are associated with immunosuppression in myeloma (23) and less Bregs could suppress IL-10 expression, thus limiting Treg expansion (24, 25). PVd treated patients showed significantly (p=0.01) less Bregs (CD19+CD43-CD5+) following treatment at day 8 of cycles 1 and 3 compared with screening, but this kind of decrease in Bregs was not seen in Vd treated patients (**Figure 4K**). Vd treated patients also had decreased populations of innate response activator B cells (IRA-B) (CD19+GM-CSF+) while PVd treated patients maintained similar cell frequencies throughout each timepoint (**Figure 4L**). In both arms, the number of MZB cells (CD19^+^CD23^+^) increased from screening through cycle 1 and cycle 3 (**Figure 4M**). At the same time, both PVd and Vd patients showed significantly decreased populations of follicular B cells (FB) (**Figure 4N**). FB cells are a subset of germinal center B cells thought to produce myeloma cell-precursors (26, 27). There was no difference in CD27+ memory B cell-populations following either treatment (**Figure 4O**).

**Figure 5:**
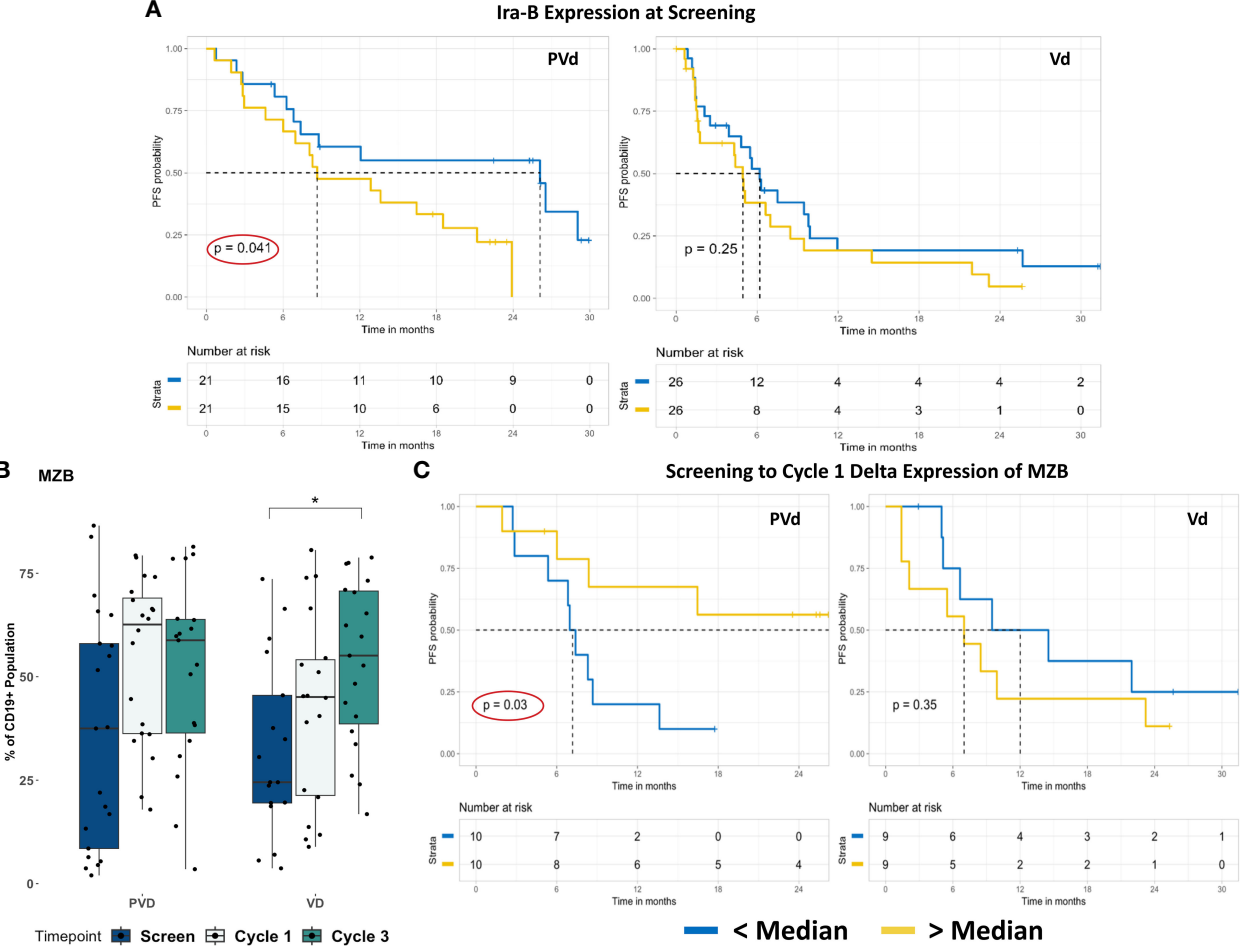
Representative screening and post-treatment B cell-sub-populations associated with favorable PFS in PVd–treated patients. Specific B cell-immune markers that are associated with PFS as shown in panel **(A–C)**. The expression of Ira-B cells, at screening in association with PFS was shown **(A)**. The expression of MZB cells population in paired samples **(B)** and their association with PFS at cycle 1 relative to screening for PVd treated patients shown **(C)**. The number of stars indicates the level of p-value significance (*p<0.05) between groups including, screening vs cycle 1; screening vs cycle 3 and cycle 1 vs cycle 3 for each marker. The Kaplan-Meier survival plots are drawn for each arm separately for the expression of a given immune marker (Ira-B and MZB) expression below median (blue dark line) and above median (yellow light line).

Next, we evaluated the differences seen in B cell-populations in terms of patient survival and determined that two different B cell-populations (Ira-B and MZB) are associated with patient survival. When we compared the frequency of CD19^+^ Ira-B cells between the two treatment groups, there was a significant decrease at day 8 of cycles 1 and 3 from screening in the Vd treatment arm, shown in **Figure 4L**. However, the PFS analysis of patients revealed that while a lower than median expression of CD19+ Ira-B cells at screening significantly (p=0.041) improves PFS for PVd treated patients (additional 21 months) in comparison with higher expression of IraB cells (above median) as shown in **Figure 5A**. On other hand, Vd treated patients had no significant difference in survival, as shown in **Figure 5A**. The increasing trend of MZB cells was observed in paired samples in both treatment arms, though it was found to be unsignificant (**Figure 5B**). More importantly, a higher than median expression of MZB cells at cycle 1 day 8 in comparison to screening for PVd treated patients exhibited significantly (p=0.03) enhanced PFS (additional 20 months) in comparison with patients who had lower than median expression of MZB. Curiously, a reverse trend in the frequency of MZB was seen in Vd treated patients (**Figure 5C**).

### Pomalidomide has varying effects across T cell subtypes and their association with clinical outcome benefit

**Figure 6:**
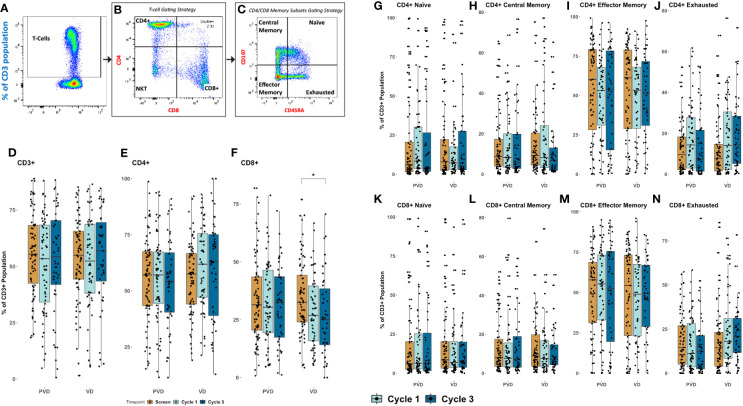
Peripheral pharmacodynamic alterations in CD3+ T cell-population following PVd and Vd treatments. **(A)** Representative dot plot analysis for gating strategy for CD3+ T cell sub-populations. **(B)** Frequencies of CD4+ and CD8+ T cells from CD3+ gating. **(C)** Memory cells, including naïve cells, central memory cells (CM), effector memory cells (EM), and exhausted cells (EX) were measured using CD45RA and CCR7. Changes in CD3+ T cells **(D)**, in CD4+ T cells **(E)**, and in CD8+ T cells **(F)** in PVd and Vd treated patients. Pharmacodynamic trends were noted in memory populations of CD4+ T cells including naïve **(G)**, CM **(H)**, EM **(I)** and EX **(J)**; of CD8+ T cells including naïve **(K)**, CM **(L)**, EM **(M)** and EX **(N)**. The number of stars indicates the level of p-value significance (*p<0.05) between groups including, screening vs cycle 1; screening vs cycle 3 and cycle 1 vs cycle 3 for each marker.

The four (4) memory T populations were evaluated by multi-color flow cytometry, including, naïve, central memory (CM), effector memory (EM), and exhausted memory (EX). Representative dot-plot gating strategy for T cell memory sub-populations is shown in **Figures 6A–C**. Initially, CD3 population were gated from the lymphocyte gate, as seen in **Figure 6A**. From the CD3 gated population, both CD4 and CD8 are sub-gated, as seen in **Figure 6B**. The four memory (CM, Naïve, EM and EX) subset populations were then gated from their respective T-cell population, CD4 or CD8, using markers CD45RA and CCR7 (CD197), **Figure 6C**. Our analysis revealed no disparity among CD3+ T cells and CD4+ helper T cells, following either treatment at their respective timepoints (**Figures 6D, E**, respectively). However, CD8+ cytotoxic T lymphocytes were significantly (p=0.05) decreased in frequency at day 8 of cycle 3 in comparison to screening among Vd treated patients (**Figure 6F**). This decrease in CD8+ T cells was not seen in PVd treated patients. Subsequent investigation of naïve populations in both CD4 (**Figure 6G**) and in CD8 (**Figure 6K**) following either treatment showed no changes. There is no change in CM and EM populations in both CD4+ (**Figures 6H, I** respectively) and in CD8+ (**Figures 6L, M** respectively) T cells in either treatment. However, we noted a consistently increasing tendency in the frequency expression of exhausted T-cells for both CD4+ and CD8+ cells in the Vd arm (**Figures 6J, N** respectively).

**Figure 7:**
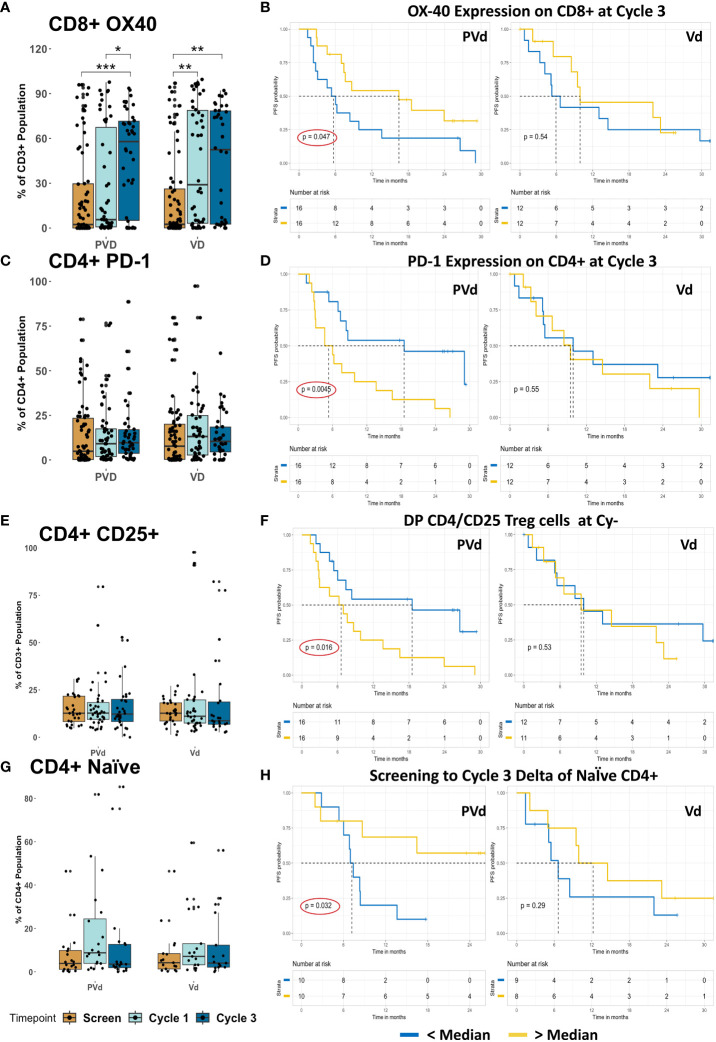
Association of PFS in patients with T cell-marker expression after PVd treatment in comparison with Vd treatment. **(A)** Showing the expression OX-40 on CD8+ T cells. **(B)** The association between the CD8+ T cell-expression of OX-40 and PFS at cycle 3. **(C)** Box graphs showing the expression of PD-1 on CD4+ T cells. **(D)** Correlation between PFS and expression of PD-1 on CD4+ T cells at cycle 3. **(E)** Presence of regulatory T cells with CD4+CD25+ following treatments. **(F)** The influence of regulatory T cells on PFS. **(G)** Box graphs showing naïve CD4+ T cells in paired samples. **(H)** Higher numbers of naïve CD4 T cells following PVd treatment increase survival in patients. The number of stars indicates the level of p-value significance (*p<0.05; **p<0.01; ***p<0.001) between groups including, screening vs cycle 1; screening vs cycle 3 and cycle 1 vs cycle 3 for each marker. The Kaplan-Meier survival plots are drawn for each arm separately for the expression of a given immune marker (OX-40 on CD8, PD-1 on CD4, CD25 on CD4, and naïve CD4)) expression below median (blue dark line) and above median (yellow light line).

One of the activation markers, OX-40, was significantly increased in its expression on CD8+ T cells following both treatments at cycle 1 day 8 and cycle 3 day 8 compared with respective screening samples, seen in **Figure 7A**. Furthermore, if the expression of OX-40 on CD8+ T cells at cycle 3 day 8 is higher than the median expression, PVd patients yielded significantly (p=0.047) better PFS (additional 10 months) than the patients with lower than the median expression of OX-40 on CD8 T cells (**Figure 7B**). We observed that there is no difference in PD-1 (CD279) expression on CD4+ T cells following either treatment compared between respective screening samples, shown in **Figure 7C**. However, when PD-1 on CD4+ T cells at cycle 3 day 8 falls below the median expression value, PFS was significantly (p=0.0045) prolonged in PVd treated patients by an additional 14 months compared to patients who had higher expression of PD-1 (above median) as seen in **Figure 7D**. This is important, as exhausted T cells are a common feature of myeloma and can interfere with the efficacy of immunotherapies (28).

Finally, there is no difference in the frequency of regulatory T cells expressing CD4+CD25+ markers following either treatment as shown in **Figure 7E**. However, after cycle 3 day 8, if regulatory T cells (CD4+CD25+) are lower than the median expression in their frequency, PVd treated patients have significantly (p=0.016) better PFS (additional 12 months) compared with patients who have higher regulatory T cells (above median) as shown in **Figure 7F**. Finally, among paired samples, there is no difference in CD4+ naïve T cells following either of the treatments as shown in **Figure 7G**. However, patients who expressed higher than median levels of CD4+ naïve T cells at cycle 3 compared to screening, were associated with improved PFS, which was dramatically significant (p=0.032) for PVd treated patients resulting in an additional 24 months of survival as seen in **Figure 7H**. These survival benefits were not seen in Vd treated patients with the above-mentioned immune markers on T cells.

## Discussion

Pomalidomide, velcade, and dexamethasone each affect the immune system in specific manner, and often in complex ways. To our knowledge, we are the first study to assess, in-depth, the effect of Vd doublet or PVd triplet therapy on 33 immune subpopulations using the OPTIMISMM trial with 366 PBMC samples from 186 patients. The OPTIMISMM trial showed both a higher response rate and an improved PFS in PVd treated patient when compared to Vd treated patients. Here we have analyzed associations between the improved outcome with the addition of pomalidomide to Vd treatment and its impact on immune milieu.

Immunomodulatory agents can favorably impact NK cell composition, both in terms of total CD16+CD56+ cells and proliferating Ki67+ NK cells (19, 29). While we found that the total number of CD56+ NK cells were lower in frequency following Vd treatment, this is not seen with PVd treatment. A higher expression of double-positive NKG2D/p46 on NK cells and a lower expression of double-positive KIR molecules (CD159a/p75 and CD158a/b) on NKT cells were observed in both PVd- and Vd-treated patients. We have observed a favorable association of PFS with NK cells expressing higher levels of activation markers and lower levels of KIR molecules after PVd treatment. Among PVd treated patients at cycle 1, patients expressing a higher number of NK cells expressing NKG2D alone (above median level) was associated with improved PFS compared with patients expressing lower levels of NKG2D (below median). In addition, when we investigated samples paired between screening and cycle 3, PFS in PVd treated patients was influenced in a favorable fashion with an increased number of NK cells that are double-positive for NKG2D and p46. Moreover, we show that the decreased number of KIR-expressing (CD158a/b or CD159a) NK cells by PVd treatment enhanced PFS. None of these immune markers mentioned above on NK cells are associated with survival of patients treated with Vd. This clearly indicates that the addition of pomalidomide to Vd treatment results in a therapeutic, anti-myeloma response by the activation of NK cells and by the reduced suppression of NK cells, both of which benefit the survival of PVd treated patients.

We observed that the PVd treatment decreased the population of Breg cells, which could decrease IL-10 production, thus limiting Treg expansion and enhancing immune function (29). Furthermore, either therapy decreased the population of FB cells, which are a subset of germinal center B cells that may produce myeloma cell precursors (21, 22). This could mean treatments can limit the re-supply of plasma cells long-term post-treatment by depleting an oncogenic progenitor population. The PVd-treated patients did not have decreased populations of B1a and IRA B cells unlike the Vd-treated patients, indicating improved innate immunity. Consistently, patients treated with PVd also expressed a higher proportion of B1b cells compared with screening. However, patients in both treatment arms had the same increase in MZB cells. PVd treatment increased the number of MZB cells at cycle 1-day 8 relative to screening for paired samples and that increase of MZB cells associated with significantly better PFS. These observations lead us to hypothesize that a pomalidomide-containing regimen could confer enhanced immunity and thus a favorable safety profile surrounding a decreased incidence of opportunistic infections.

PVd treatment did not result in a reduction of CD8 cell-numbers, as is seen in Vd treated patients. It appears that the addition of pomalidomide to Vd treatment increased the number of CD8+ cells expressing OX-40, which is a co stimulatory molecule, and this increase by PVd is favorably associated with PFS. On the other hand, PVd treatment decreased the number CD4+ cells expressing PD-1, enhancing the overall anti-myeloma response, and subsequently PFS was improved. Finally, downregulating the number regulatory T cells by PVd treatment prolongs survival in patients as well.

Immune enhancements in the T cell memory subpopulation and increases in proliferating NK cells are caused by two newer IMiDs agents, iberdomide and CC-92480 (30, 31). Future studies should focus on more granular definitions of B cell and NK/NKT subpopulations using the subpopulations defined in this study. In addition, while the BM data was not available for our analysis in this study, it should be included in future studies to provide deeper contextual meaning, as it did in the study of iberdomide (32). When the cultured B cells from systemic lupus erythematosus (SLE) patients in the presence or absence of iberdomide, it reduced the B cells ability to produce auto antibodies (33). Using *in-vitro* culture models, iberdomide was able to stimulate T cells isolated from healthy donors. These T cells produced IL-2 when they were stimulated by anti-CD3 antibody, and they also produced lower levels of IL-1-beta when stimulated by LPS. These results indicate that the hallmark characteristics of SLE, including auto-antibody production, regulatory T cell-dysfunction and inflammatory responses, are inhibited by iberdomide. In this context, iberdomide may be improving immune function by reducing systemic regulatory T cells. Previously, we have already shown similar types of auto-immune characteristics including dysfunctional regulatory T cells in myeloma (34). Our results in this study with PVd also support the concept that a reduction of regulatory T cells following treatment is associated with favorable PFS when compared with patient expressing higher levels of regulatory T cells. Immune modulatory drugs like lenalidomide and pomalidomide have shown to reduce the influence of regulatory T cells (35). Another recent study demonstrated that lower levels of MZB contributed to SLE pathology (36) and in this current study, we showed that an increased frequency of MZB is associated with better PFS in PVd treated patients. The addition of pomalidomide to Vd treatment may play a role in controlling auto-immune phenomenon in myeloma patients by reducing regulatory T cells and increasing MZB cells. The ability of these immune modulatory agents, like lenalidomide and pomalidomide, to reduce auto-immune characteristics in myeloma patients’ needs to be further evaluated in future studies.

**Figure 8:**
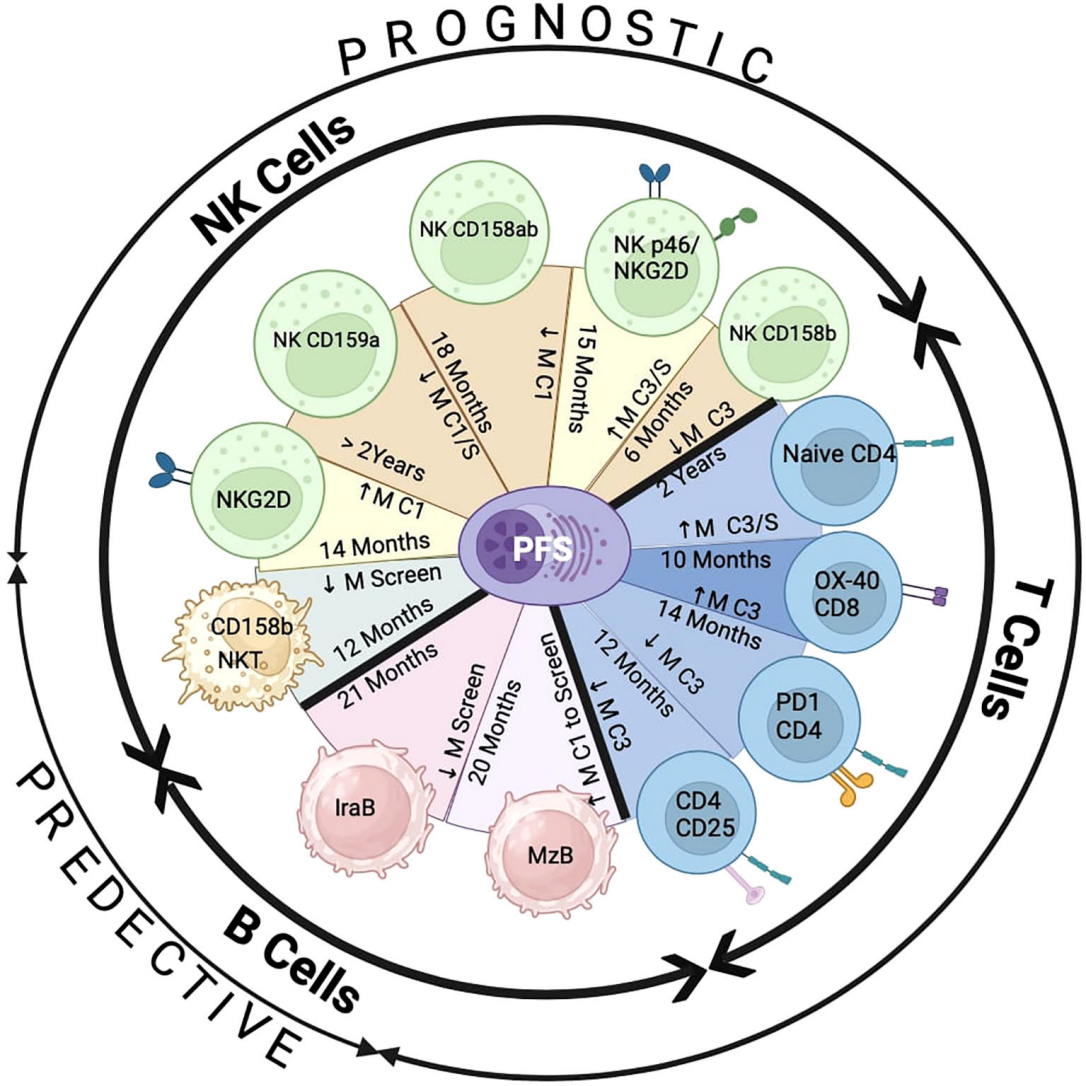
Summary of PFS association with the expression of immune markers by PVd treatment. There are two predictive markers at screening – expression of CD158b on NKT cells and expression of IraB in B cells. There are ten prognostic immune markers by PVd treatment – five on NK cells (NKG2D, CD159a, CD158a and b, p46 and NKG2D, CD158b), four on T cells (Naïve CD4, OX-40 on CD8, PD-1 on CD4, CD25 on CD4 regulatory T-cells) and one on B cells (MZB). ↓ M=Less than median expression. ↑ M=Greater than Median expression. C1=Cycle 1 Day8. C1/S= Cycle 1 Day 8 in comparison with screening. C3=Cycle 3 Day 8. C3/S= Cycle 3 Day 8 in comparison with screening. This figure was created using the Bio-Render program.

We summarized in **Figure 8**, the influence of the addition of pomalidomide to Vd therapy by improving PFS with alterations in the number of immune biomarkers, at screening (predictive) and at cycle 1/3 (prognostic), on NK/NKT, B, and T-cells. We showed a predictive marker (CD158b) on NKT cells in association with PFS due to PVD treatment. Additionally, expression higher than median compared to lower than median expression of two activation prognostic markers (NKG2D alone and p46/NKG2D) and lower than median compared to higher than median expression of three inhibitory prognostic markers (CD159a, CD158a/b and CD158b alone) on NK cells showed significantly enhanced PFS by PVD treatment. These results indicate that the addition of pomalidomide to Vd treatment improved patients’ survival by reducing inhibitory molecules and by activating NK cells. We have shown a predictive marker (Ira-B) and prognostic marker (MZB) in the B cell-compartment. The addition of pomalidomide may control inflammatory burden by enhancing innate immunity through a reduction of Ira-B cells and upregulate immunity against encapsulated bacterial infections by enhancing MZB. We have shown four prognostic markers (Naïve CD4, OX-40+ CD8, PD-1+ CD4 and CD25+CD4). These results indicate that the addition of pomalidomide to Vd treatment may improve clinical response and patient’s survival by increasing naïve CD4+, and OX-40+ CD8+ T cells; and by reducing PD-1+ CD4+ and regulatory T cells. In summary, the prognostic significance of immune markers on PFS was only observed in the PVd treated patients, and none of the immune markers results in significant prognostic value in the Vd-only arm. Thus, this study exhibits the importance of the immunomodulatory effects for therapeutic benefit by adding pomalidomide to Vd treatment.

## Data availability statement

The original contributions presented in the study are included in the article/supplementary material. Further inquiries can be directed to the corresponding authors.

## Ethics statement

The studies involving humans were approved by Dana-Farber Cancer Institute, Harvard Medical School. The studies were conducted in accordance with the local legislation and institutional requirements. The participants provided their written informed consent to participate in this study.

## Author contributions

RP: Conceptualization, Data curation, Funding acquisition, Investigation, Methodology, Project administration, Resources, Software, Supervision, Validation, Visualization, Writing – original draft, Writing – review & editing. WP: Conceptualization, Funding acquisition, Project administration, Visualization, Writing – original draft, Writing – review & editing. MS: Visualization, Writing – original draft, Conceptualization, Data curation, Formal analysis, Investigation, Methodology, Software, Validation. LP: Conceptualization, Data curation, Formal analysis, Methodology, Software, Validation, Visualization, Writing – original draft. KH: Data curation, Methodology, Resources, Software, Writing – original draft. TP: Data curation, Methodology, Resources, Software, Writing – review & editing. ST: Data curation, Formal analysis, Methodology, Software, Validation, Writing – review & editing. AW: Data curation, Methodology, Writing – review & editing. AKa: Data curation, Methodology, Writing – review & editing. SV: Data curation, Methodology, Writing – review & editing. MB: Data curation, Methodology, Software, Writing – review & editing. VB: Data curation, Methodology, Writing – review & editing. HS: Data curation, Methodology, Writing – review & editing. AKr: Data curation, Methodology, Writing – review & editing. SD: Data curation, Formal analysis, Methodology, Software, Writing – review & editing. MF: Conceptualization, Data curation, Methodology, Resources, Writing – review & editing. SL: Data curation, Methodology, Software, Writing – review & editing. PR: Conceptualization, Resources, Writing – review & editing. KA: Conceptualization, Investigation, Project administration, Resources, Writing – review & editing. JC: Conceptualization, Investigation, Resources, Writing – review & editing. HA-L: Conceptualization, Resources, Writing – original draft. AT: Conceptualization, Funding acquisition, Investigation, Project administration, Resources, Supervision, Visualization, Writing – original draft. NM: Conceptualization, Funding acquisition, Investigation, Project administration, Resources, Supervision, Visualization, Writing – original draft.
